# A Sulphur Bath

**Published:** 1878-10

**Authors:** 


### A SULPHUR BATH.

Greene came into the office one day, sat nervously moving his knees in imitation of the armature of a telegraph instrument, and while he detailed the symptoms that abide with the dyspeptic, continually rubbed the cuticle at the junction of his fingers.

"But, what is the trouble with your fingers?" we inquired, after his other ailments had been duly discussed. "You seem to have some irritation between them."

He ceased his blissful scratching for a moment and held his papular fingers up for inspection complacently remarking:

"Bless me if I know; they have been itching like fury for a week, and the only relief I have found is to scratch."

"Why, man, you have the itch!"

"Itch!" he exclaimed, rising to his feet and wiping the cold perspiration from his forehead. "Itch! where in the canopy could I have obtained that?"

"Really, my dear sir, that query I am unable to satisfy, but I can certify that you are in possession of as handsome an itch as ever came under my observation."

"What shall I do to get rid of it?" he anxiously inquired, holding his two hands out before him, digits widely spread, and inspecting closely the little vesicles with which they were studded.

"You must take a sulphur bath."

"How?"

"Well go down street and procure a tight dry-goods box, one that will admit of your person seated upon a stool, so that your neck will come even with the top of the box. We will then improvise a sulphur bath that will destroy the itch mites at one sitting."

In an instant he was gone, soon returning with the box upon the shoulder of a stalwart drayman. It was placed in a closet, the victim seated inside, the lid made to encircle his neck, the cracks all carefully plugged, and a large alcohol lamp with a dish of sulphur over the flame, placed near his feet.

Soon the fumes of sulphur began to stream through the crevices, poured through the space about his neck, got into his nostrils and filled his eyes, until he coughed, sneezed and wheezed like a windbroken horse. The sweat oozed from his skin and formed little rivulets down his back running through the bottom of the box to make a pool upon the floor outside. All this, he stood bravely —yes, we can put it stronger and say marvelously.

"How long must I sit here?" he gasped, at the end of a half hour.

"O stand it for an hour longer," we replied. "It is a sure cure for the itch, my boy!"

This last remark seemed to console him, while the sulphur fumes as surely were suffocating him.

We went to the closet to be certain that he was still alive. His face was as red as a boiled lobster, great beads of perspiration dropped from his nose and chin, while the poor fellow turned his head in various directions to escape the constantly increasing avenues for the exit of the sulphurous vapors. Wheezing, coughing and squirming, he opened one eye, looked imploringly toward us and gasped:

"Am—I—not—done—enough?"

"Nearly—ten minutes longer, and you will be cured, if alive!"

We sat down by an open window to secure a breath of fresh air, and waited.

Instantly, a report as from a cannon rang in our ears as Greene fell upon the floor, coming through the closet door as though fired from a mortar. He gathered himself up, and as he stood under the brilliant chandelier, he presented the appearance of the traditional singed cat. The alcohol lamp had exploded, and hair, whiskers, eyebrows and eyelashes of the patient were burned short; their bristling, stubby ends only marking the positions where hair once abounded.

"That's a thundering good thing!" he meekly said, as he stood surveying himself and shaking the crisp hair from his head: "Why didn't you suggest it before?" I've been all over the world,—taken all kinds of baths,—been in all sorts of infernal places,—but this itch bath of yours takes the belt for novelty of situation and abruptness of termination. Cure! I'll guarantee it to cure anything. I know a fellow that's love-sick,—got it awfully bad, writes poetry, doesn't eat, and all that; he shall take this bath to-morrow, even if I sleep with him to-night that he may have a share of my itch. Cure? Of *course* it will cure."

Then he wandered into the next room, in search of his clothing—groping his way along and muttering something about Vesuvius—and a supposed-to-be warmer country, not polite to mention.

